# Aminophylline restores glucocorticoid sensitivity in a guinea pig model of sudden sensorineural hearing loss induced by lipopolysaccharide

**DOI:** 10.1038/s41598-017-02956-x

**Published:** 2017-06-02

**Authors:** Qiong-Qiong Zhou, Yan-Hong Dai, Xiao-Ping Du, Jie Hou, Hui Qi, Wan-Dong She

**Affiliations:** 1 Department of Otolaryngology-Head and Neck Surgery, Nanjing Drum Tower Hospital, Medical School of Nanjing University, Nanjing, 210008 China; 2 Hough Ear Institute, Oklahoma city, Oklahoma, 73112 USA; 3 Department of Otorhinolaryngology, Children’s Hospital of Nanjing Medical University, Nanjing, 210008 China

## Abstract

Glucocorticoids have been used to treat hearing loss and vestibular dysfunction for many years. However, some reports have indicated that a subset of patients with these disorders exhibit glucocorticoid insensitivity or resistance. A reduction in histone deacetylase 2 (HDAC2) activity and expression has been reported to play a critical role in glucocorticoid resistance. Here, we investigated the protective effects of aminophylline on HDAC2 expression and glucocorticoid sensitivity in lipopolysaccharide (LPS)-induced sudden sensorineural hearing loss in guinea pigs. We assessed hearing recovery in LPS-applied guinea pigs, which were either left untreated or were systemically treated with either dexamethasone, aminophylline, or a combination of the two. We utilized fluorescence microscopy and enzyme-linked immunosorbent assay to analyze the distribution patterns of HDAC2 and detect its levels in the cochlea. We used hematoxylin-eosin staining to examine cochlear histopathological changes. In the absence of treatment, significant hearing loss was detected in LPS-exposed animals. A synergistic effect was observed between aminophylline and dexamethasone in maintaining HDAC2 expression levels, preventing hearing loss in LPS-exposed animals and reducing cochlear damage. This study indicates that aminophylline can restore glucocorticoid sensitivity, which provides a new approach to treating patients with hearing disorders who are refractory to glucocorticoids.

## Introduction

It has been 60 years since glucocorticoid therapy was first used to treat hearing and balance disorders, such as sudden idiopathic sensorineural hearing loss (SSNHL), autoimmune inner ear diseases, and Meniére’s disease^1–3^. Glucocorticoid therapy has also been used to control inflammation in the inner ear induced by otitis media or bacterial meningitis^4, 5^. Although the molecular mechanisms underlying glucocorticoid treatment are not well characterized, it is believed that glucocorticoids suppress inflammation and pathological immune responses in the inner ear^6, 7^. Glucocorticoids bind to glucocorticoid receptors (GRs) and recruit histone deacetylase 2 (HDAC2) to switch off multiple inflammatory genes that encode for cytokines, chemokines, adhesion molecules, and inflammatory enzymes by repressing nuclear factor-κB (NF-κB), a pro-inflammatory transcription factor^8, 9^. Despite the numerous benefits of glucocorticoid treatment for hearing and vestibular dysfunction, some clinical reports and reviews indicate that a subset of patients with these disorders do not respond to glucocorticoid treatment. In other words, these patients exhibit glucocorticoid insensitivity or resistance^10, 11^.

Among these patients, the molecular mechanisms of glucocorticoid resistance have not been clearly elaborated. However, recent studies have demonstrated that reduced HDAC2 activity and expression plays a critical role in glucocorticoid insensitivity or resistance^8, 9^. Inflammation has been shown to impair HDAC2 activity, which may, for instance, contribute to the glucocorticoid insensitivity associated with chronic obstructive pulmonary disease (COPD)^9, 12^. This rationale is supported by the fact that activation of NF-κB by inflammation and oxidative stress is also associated with glucocorticoid insensitivity^13^. In our previous studies, we discovered that this dynamic interplay may also influence treatment strategies for SSNHL, as we observed that the effectiveness of glucocorticoid application was positively correlated with HDAC2 levels^14^.

Theophylline, a hydrophilic methylxanthine derivative, has been shown to play an important role in inhibiting inflammation through increasing the expression level and activity of HDAC2^15^. Aminophylline (AMI) is a formulation of theophylline with ethylenediamine in a 2:1 ratio for enhanced solubility. The aims of this study were to demonstrate the relationship between HDAC2 levels and glucocorticoid responsiveness and to explore the potential effects of AMI in restoring glucocorticoid sensitivity in the cochleae of guinea pigs in an established animal model of SSNHL induced by intracochlear injection of lipopolysaccharide (LPS), at endotoxin levels which have been shown to cause rapid and pronounced auditory brainstem response (ABR) threshold shifts^16, 17^.

## Results

### AMI improves the protective efficacy of glucocorticoids against hearing loss induced by LPS

**Figure 1:**
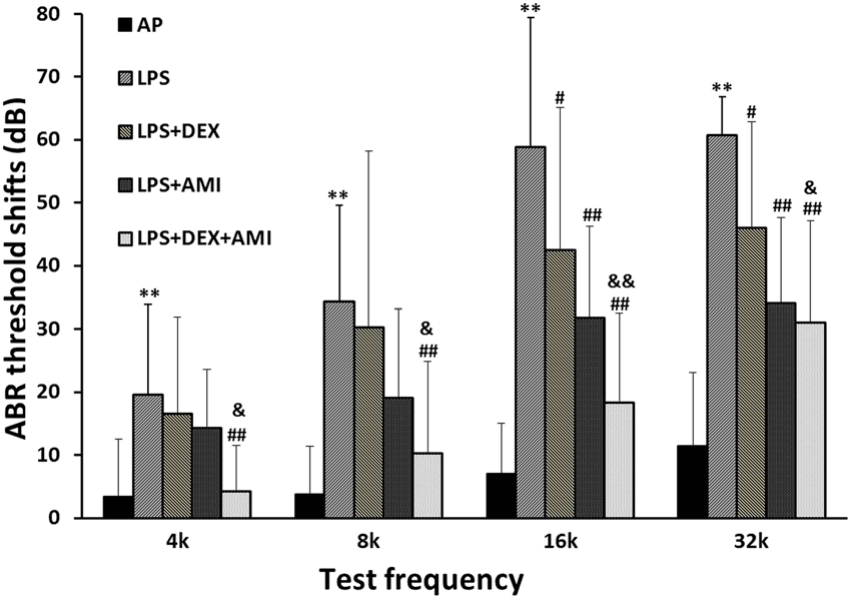
ABR threshold shifts (dB SPL) 48 h after surgery in each group. AP infusion alone did not cause significant ABR threshold shifts. In contrast, cochlear LPS infusion significantly induced hearing loss at all frequencies tested in guinea pigs. Smaller threshold shifts were observed in the LPS + DEX or LPS + AMI group compared to the LPS group, especially at high frequencies (16 and 32 KHz). However, the combination of DEX and AMI showed a significant protective effect against LPS-induced hearing loss at all frequencies compared to both the untreated LPS group or the LPS + DEX group at all frequencies tested. There were ten animals in each group. * and ** indicate *p* < 0.05 and *p* < 0.01 compared to the AP group, respectively; ^#^ and ^##^ indicate *p* < 0.05 and *p* < 0.01 compared to the LPS group; ^&^ and ^&&^ indicate *p* < 0.05 and < 0.01 compared to the LPS + DEX group, respectively.

In support of our surgical approach and specificity of LPS-induced sensorineural hearing loss (SNHL), cochlear infusion of the vehicle, artificial perilymph (AP), alone did not cause significant ABR threshold shifts (less than 10 dB on average). In contrast, cochlear LPS infusion specifically induced hearing loss in guinea pigs, such that a greater elevation of threshold shifts was observed in the untreated, LPS-infusion group at all frequencies tested. The LPS-induced SNHL manifested in a basal-to-apical gradient, with the most pronounced loss observed at high frequencies (19.5 ± 14.33, 34.25 ± 15.28, 58.75 ± 20.66 dB, and 60.75 ± 6.02 for 4, 8, 16, and 32 kHz, respectively). However, smaller threshold shifts were observed among guinea pigs treated with dexamethasone (DEX) or AMI. The ABR threshold shifts in both the LPS + DEX group and the LPS + AMI group were smaller than those measured in the untreated, LPS group, with statistically significant differences detected at 16 kHz (42.5 ± 22.64 dB, *p* = 0.036 and 31.75 ± 14.53 dB, *p < *0.01, respectively) and 32 kHz (46 ± 16.80 dB, *p* = 0.018 and 34 ± 13.60 dB, *p* < 0.01, respectively). Although smaller threshold shifts were observed in the AMI-treated group than in the DEX-treated group at higher frequencies (8, 16, 32 kHz, Fig. 1), these differences were not statistically significant across all frequencies tested (all *p* > 0.05). However, the combination of DEX and AMI showed a significant protective effect at all frequencies tested (4.25 ± 7.27, 10.25 ± 14.55, 18.25 ± 14.29, 31 ± 16.08 dB for 4, 8, 16, 32 kHz, respectively) against the LPS-induced hearing loss compared to either the untreated LPS group (all *p* < 0.01) or the LPS + DEX group (*p* = 0.022, 0.013, 0.002 and 0.016 for 4, 8, 16 and 32 kHz, respectively). Surprisingly, there was no significant difference between LPS + DEX + AMI and LPS + AMI groups in ABR threshold shifts at all frequencies tested (all *p* > 0.05), although there was a tendency of smaller threshold shifts at lower frequencies (4–16 kHz) in the LPS + DEX + AMI group (Fig. 1). These results suggest that AMI in the combinatorial formulation can help potentiate the protective effects of DEX against hearing loss in this animal model.

### HDAC2 is extensively distributed in the cochlea

**Figure 2:**
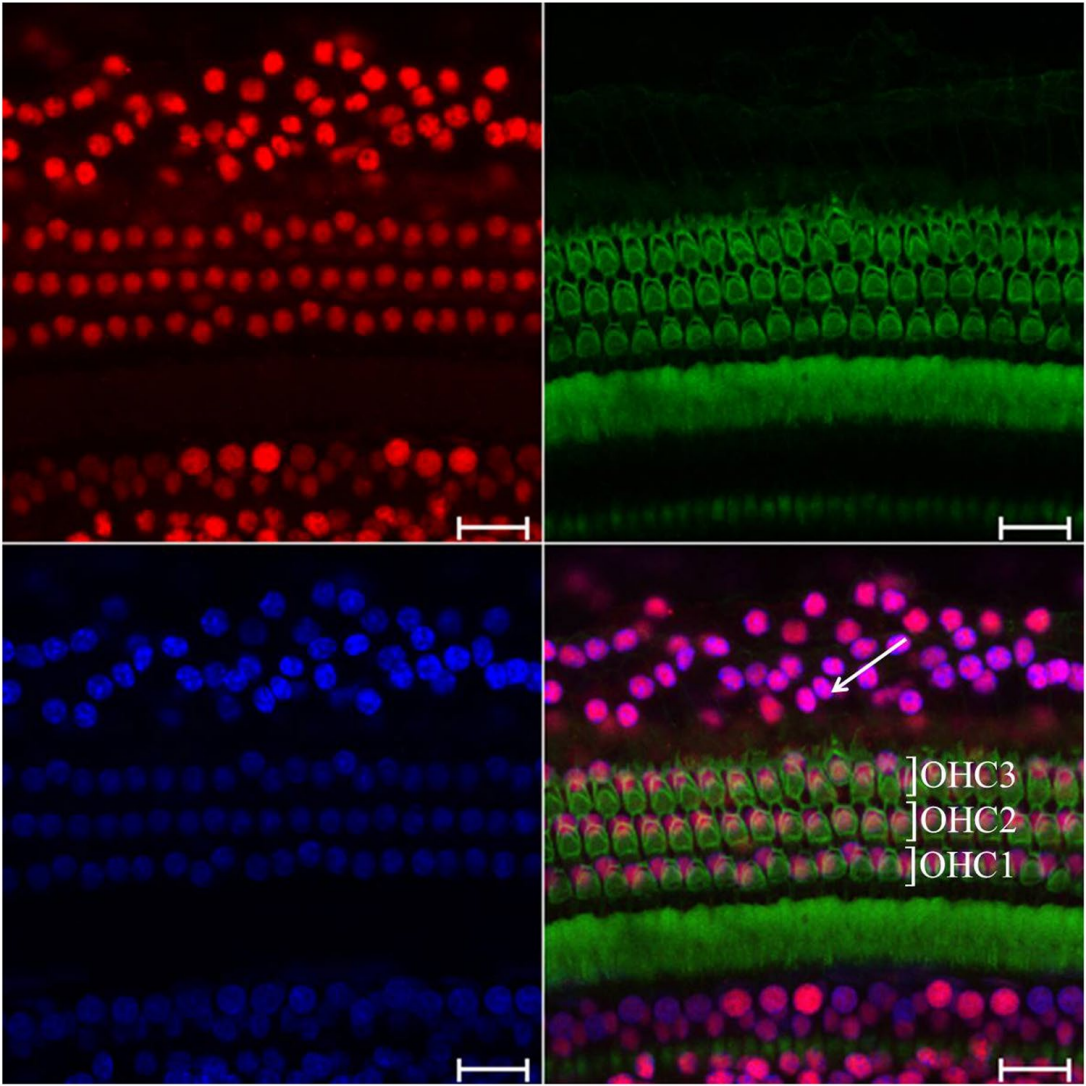
Examples of HDAC2 immunolabeling in the cochlea by whole-mount technique. FITC-conjugated phalloidin (green) and DAPI (blue) were used to label stereocilia and nuclei, respectively. Positive nuclear immunolabeling of HDAC2 (red) was observed in inner hair cells (IHCs), outer hair cells (OHCs) and Hensen cells (arrow). Scale bars = 20 μm.

**Figure 3:**
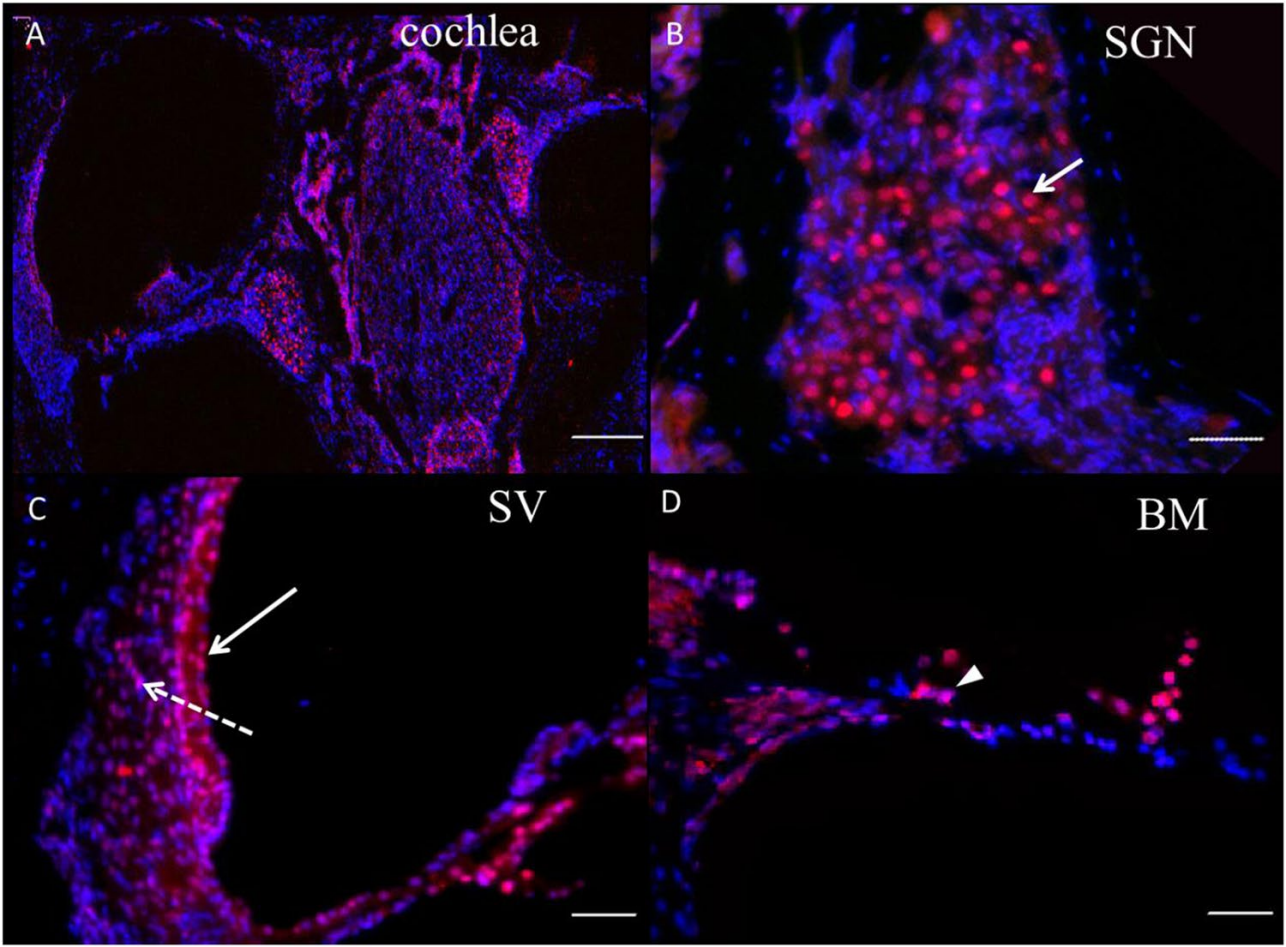
Examples of HDAC2 immunolabeling in cochlear cryosections. Low magnification of HDAC2 immunolabeling is shown in A. Strong HDAC2 positive labeling (red) was detected in the spiral ganglion neurons (SGN, *arrow* in B), in the stria vascularis (SV*, long arrow* in C) and in the organ of Corti (*arrowhead* in D). Most cells in the spiral ligaments (*dashed arrow*), in the basilar membrane (BM) and in the spiral lamina (in D) showed weak positive labeling. The nuclei were stained by DAPI (blue). Scale bars = 250 μm in A; = 50 μm in B–D.

HDAC2 expression was broadly observed in the cochleae of untreated guinea pigs. Cells with HDAC2-positive staining included inner hair cells, outer hair cells, and supporting cells. Moreover, HDAC2 co-localized with nuclear DAPI staining (Fig. 2). This result indicates that HDAC2 is localized to the nucleus of cochlear cells. Strong nuclear HDAC2 immunolabeling was also observed in spiral ganglion neurons (SGNs), cells in the organ of Corti and in the stria vascularis. Most cells in the spiral ligament and in the basilar membrane also showed positive labeling (Fig. 3). We did not observe changes in the HDAC2 distribution pattern in cochleae from the LPS and drug treated groups compared to the normal controls.

**Figure 4:**
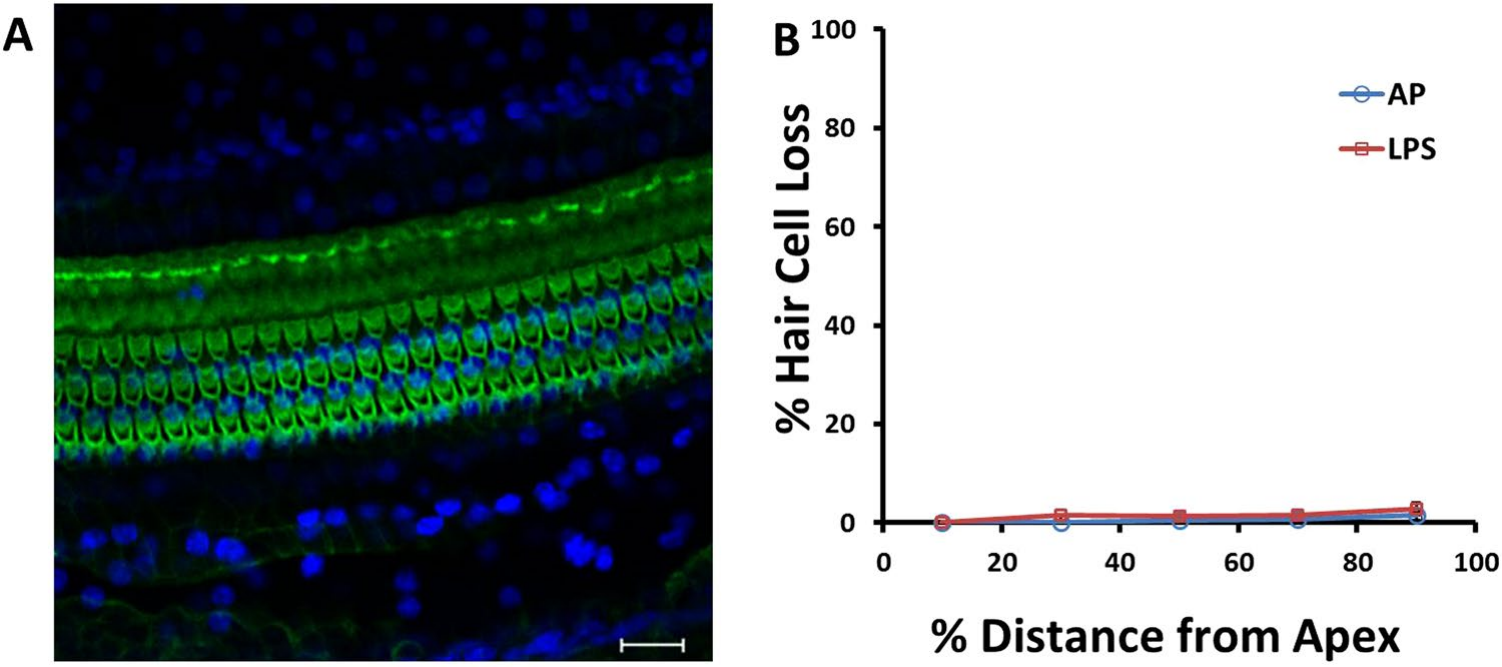
Effect of LPS on hair cells. (**A**) Example of hair cell stereocilia labeling in the LPS group. FITC-conjugated phalloidin (green) and DAPI (blue) were used to label stereocilia and nuclei, respectively. No hair cell loss was observed in the organ of Corti. Scale bar = 20 μm. (**B**) No significant hair cell loss in the LPS group compared to the AP group.

The stereocilia of hair cells were also stained with FITC-conjugated phalloidin. The morphology of stereocilia and nuclei in the organ of Corti were examined using a fluorescent microscope. However, no change was observed in the morphology of stereocilia or nuclei in the organ of Corti in either LPS (Fig. 4A) compared to the group injected with the AP vehicle alone. No significant hair cell loss was observed in the LPS group compared to the AP group (p > 0.05, Fig. 4B).

### HDAC2 levels in the cochlea

**Figure 5:**
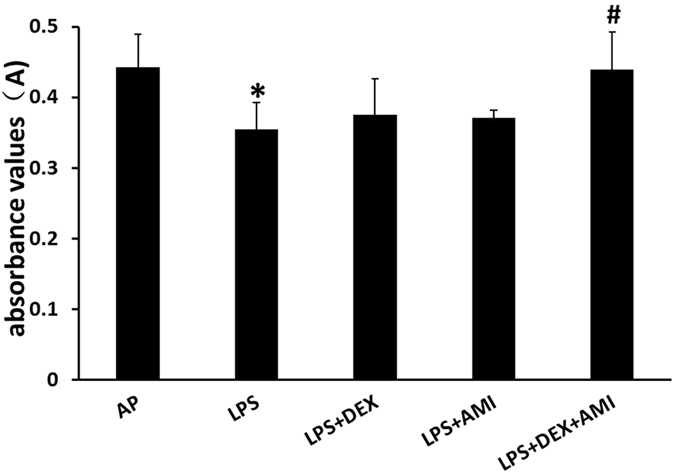
The combination treatment of aminophylline and dexamethasone mitigates ototoxic reductions of HDAC2 levels induced by LPS in the cochlea. Guinea pigs treated with LPS only had a significantly lower level of HDAC2 in cochlear tissues compared to the AP group (*p < 0.5). The level of HDAC2 was significantly increased after treating with a combination of DEX and AMI compared to the untreated, LPS-exposed group (^#^ *p* *<* 0.05). However, no significant change in the level of HDAC2 was observed in the LPS + DEX or LPS + AMI groups compared to the LPS only group (all *p* > 0.05).

While we did not observe marked differences in the HDAC2 localization patterns in treated and untreated LPS-exposed cochleae, discerning changes in the relative expression (i.e. intensity of fluorescent immunolabeling) is difficult to accurately quantify and is often subjective. Therefore, we measured HDAC2 levels in the cochlea by a more quantitative and objective enzyme-linked immunosorbent assay (ELISA) approach. Using this method, we found that guinea pigs treated with LPS alone had a significantly lower level of HDAC2 in cochlear tissues compared to the AP only group (0.354 ± 0.039 versus 0.442 ± 0.047, *p* = 0.032), indicating that LPS inhibited HDAC2 expression in the cochlea. However, no significant difference was found between either the LPS + DEX group (0.376 ± 0.051) and the LPS only group (*p* > 0.05) or between the LPS + AMI group (0.371 ± 0.011) and the LPS only group (*p* > 0.05). However, cochlear HDAC2 levels were found to be significantly increased in animals treated with the combination of DEX and AMI compared to the untreated LPS group (0.439 ± 0.054 versus 0.354 ± 0.039, *p* = 0.038, Fig. 5). These results indicate a synergistic effect between AMI and DEX in increasing HDAC2 levels in the cochlea.

### Correlation between hearing loss and cochlear HDAC2 levels

**Figure 6:**
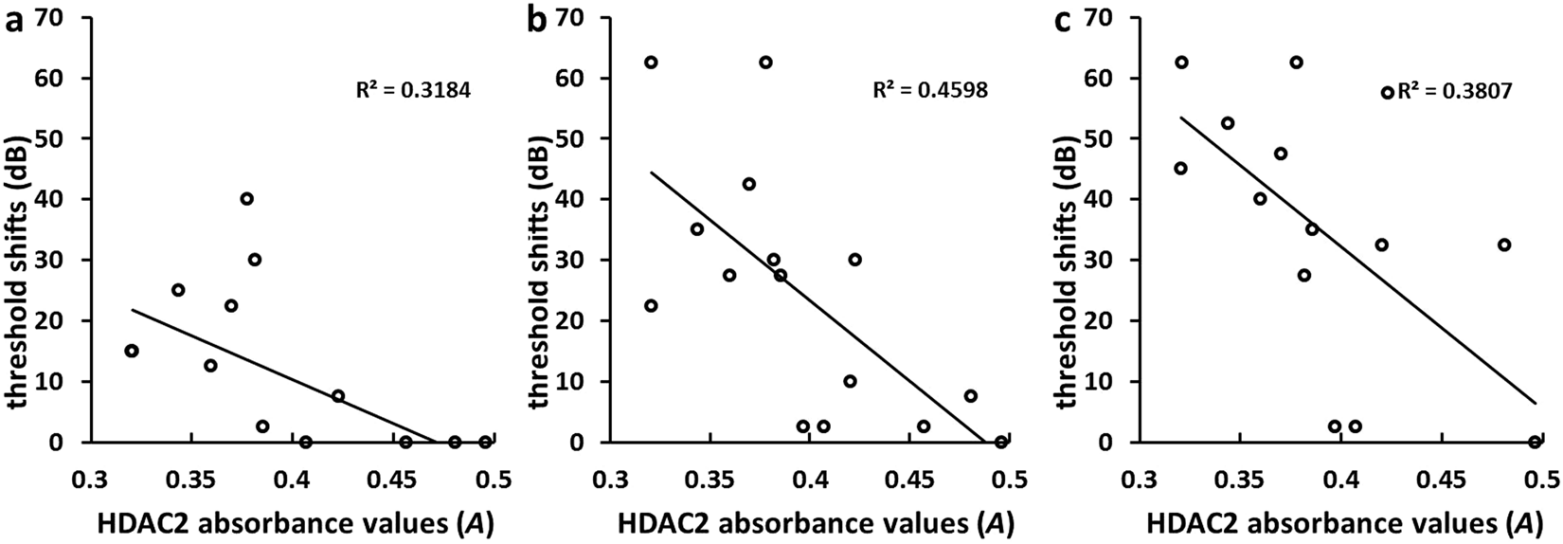
Correlation between ABR threshold shifts at different frequencies and HDAC2 levels in the cochlea. Negative correlation was observed at 8 kHz ((**a**), correlation coefficient *r* value = −0.56, *p* < 0.05), 16 kHz ((**b**), *r* value = −0.68, *p* < 0.05) and 32 kHz ((**c**), *r* value = −0.62, *p* < 0.05).

Correlation analyses between hearing loss and HDAC2 levels were also examined. The results from these analyses revealed that HDAC2 levels in cochlear tissues were negatively correlated with ABR threshold shifts at 8, 16, 32 kHZ (*r* = −0.56 and *p* = 0.028 in Fig. 6a, *r* = −0.68 and *p* = 0.005 in Fig. 6b, *r* = −0.62 and *p* = 0.014 in Fig. 6c, respectively). However, no correlation was observed between the level of HDAC2 and threshold shift at 4 kHz (*r* = −0.38, *p* > 0.05).

### AMI combined with DEX reduced cochlear damage induced by LPS

**Figure 7:**
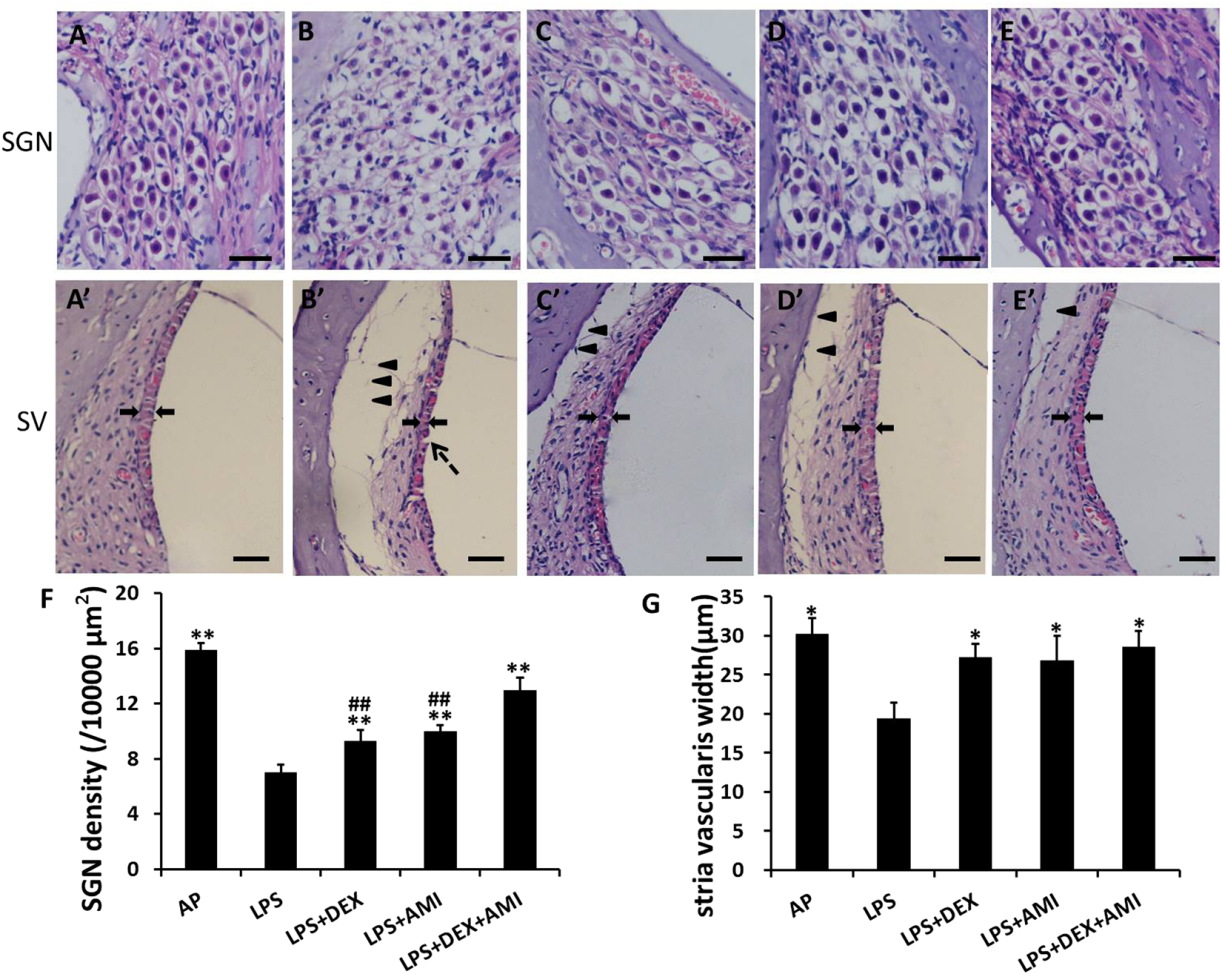
Histopathological evaluations of cochlear modiolus sections in each group. Examples of histopathological changes in the spiral ganglion (A–E) and the stria vascularis (A’–E’) in each group (HE staining). A normal spiral ganglion morphology was observed in the AP group; SGNs were orderly arranged (A). Vacuolar degeneration and disordered arrangement of the spiral ganglion cells were observed in the untreated LPS group, the number of cells in the ganglion was reduced, and the intercellular space was widened (B). Slight degeneration of the spiral ganglion was observed in the LPS + DEX (C) and LPS + AMI (D) groups. The combination of DEX and AMI reduced the degeneration of SGNs (E). A thinner stria vascularis (indicated by arrows in B’) was observed in LPS group compared to either the AP group or the active treatment groups (A’, C’–E’). Separation of the spiral ligament from the lateral walls was observed in all LPS-treated groups (indicated by arrowhead in B’–E’). However, DEX and/or AMI treatment reduced this separation (C’–E’). The dashed arrow indicates fracture of the stria vascularis, which was only observed in the untreated LPS group (B’). Scale bars = 50 μm in A–E and A’–E’. SGN cell density (F) and stria vascularis width (G) measurements were conducted for each experimentalgroup. A significantly lower SGN density was observed in the LPS group compared to the AP, AMI and/or DEX treated groups. A significantly thiner stria vascularis was observed in the LPS group compared to the AP-, AMI- and/or DEX-treated groups. * and ** indicate *p* < 0.05 and *p* < 0.01 compared to the LPS group, respectively; ^##^indicates *p* *<* 0.01 compared to the LPS + DEX + AMI group.

Local LPS application could induce serious cochlear morphological injury, including decreased spiral ganglion cell density and damage to the stria vascularis^18, 19^. In the present study, at 48 hours after LPS treatment, vacuolar degeneration and disordered arrangement of the spiral ganglion cells were observed in the untreated LPS group, such that the number of cells in the spiral ganglion was reduced and the intercellular space was widened (Fig. 7B). However, a lesser degree of degeneration of the spiral ganglion was observed in the LPS + DEX (Fig. 7C) and LPS + AMI (Fig. 7D) groups. In LPS-exposed animals treated with a combination of DEX and AMI, the degenerative effect was further reduced (Fig. 7E). The number of spiral ganglion cells was counted, and SGN densities (number of cells/10000 µm^2^) in each group was calculated (Fig. 7F). The mean spiral ganglion cell density in the untreated, LPS-exposed group (7.00 ± 0.60) was markedly reduced relative to the AP control group (15.89 ± 0.51). However, all three treatment groups exhibited average spiral ganglion cells densities (9.28 ± 0.79 in the LPS + DEX group, 9.95 ± 0.48 in the LPS + AMI group, and 12.94 ± 0.92 in the LPS + DEX + AMI group) that were significantly greater (all *p* < 0.01) than that measured in untreated controls. Nonetheless, treatment with a combination of DEX + AMI resulted in significantly greater protection against SGN loss than either treatment alone (*p* < 0.01).

Forty-eight hours after LPS perfusion in the inner ear, a thinner stria vascularis (indicated by arrows in Fig. 7B’) was observed in the untreated, LPS-exposed group compared to mock-exposed controls (Fig. 7A’). The mean width (μm) of the stria vascularis in the untreated, LPS-exposed group (19.34 ± 2.06 μm) was significantly smaller (all *p* < 0.05) than undamaged controls (30.13 ± 2.06) and each of the LPS-exposed groups that were subsequently treated with DEX, AMI, or DEX + AMI. However, there was no statistical difference in width of the stria vascularis among the DEX, AMI, or DEX + AMI treatment groups (28.56 ± 2.06, 27.21 ± 1.70, 26.76 ± 3.19 μm, respectively, all *p* > 0.05), indicating a comparable degree of protection for each of these therapeutic strategies (Fig. 7G). Separation of the spiral ligament from the lateral walls was observed in all LPS-treated groups, irrespective of treatment (Fig. 7B’–E’), however therapeutic intervention reduced this aberrant morphological effect in all cases (Fig. 7C’–E’). Fracturing of the stria vascularis was only observed in the LPS group (Fig. 7B’). No inflammatory exudate was observed in the cochlea after LPS injection (Figs 3 and 7).

## Discussion

It is known that middle ear or cochlear application of LPS causes profound inflammation, increased permeability of the blood-labyrinth barrier, decreases in cochlear blood flow, and hair cell apoptosis in the inner ear^18–21^. LPS also causes mitochondrial damage in the stria vascularis^18^. In the present study, when LPS was directly infused into the scala tympani, hearing loss was detected in all animals at all frequencies tested, especially in the high frequency regions (Fig. 1), which is consistent with previous reports^22, 23^. Meanwhile, cochlear morphological damage was observed in HE staining sections (Fig. 7). Spiral ganglion cell density was significantly decreased, and the stria vascularis was thinner in untreated, LPS-exposed animals. However, no obvious hair cell loss was observed under these conditions, and the morphology of hair cell stereocilia remained intact in phalloidin-labeled cochlear basilar membrane preparations (Fig. 4). One explanation for the measured hearing loss is the LPS-induced neural degeneration observed in the spiral ganglion. We also noted that the spiral ganglion and the stria vascularis were aberrantly altered under these pathological conditions. Thus, the electrophysiological function of the organ of Corti may be compromised 48 hours after LPS cochlear infusion as the result of disrupted ion homeostasis in the endolymph and/or energy failure in the inner ear as suggested by a previous study^24^. However, cochlear AP infusion did not cause significant cochlear damage, since threshold shifts in the AP group were minor (< 10 dB), except at 32 kHz (11.50 ± 11.56 dB), relative to the LPS-infused ears, indicating that the infusion paradigm and surgical approach were not the primary determinants for the SNHL. Alternatively, a 48 hour-interval post-LPS cochlear infusion may be too short to detect hair cell loss, even though their inherent functionality has been compromised, or perhaps, the effects were primarily manifested among the fragile ribbon synapses that innervate hair cells and whose direct examination were beyond the scope of this study. Thus, a longer term study, e.g. 1–2 weeks after the surgery, or an expanded immunohistological approach may be needed to more accurately examine the extent of hair cell injury induced by this ototoxic insult.

Development of many hearing and vestibular diseases are associated with inflammation or oxidative stress, such as autoimmune inner ear disease and SSNHL^25, 26^. For this reason, glucocorticoids are most commonly used to treat these inner ear diseases^27^. Unfortunately, about 20% of patients have no response to this treatment^28^. The mechanisms underlying glucocorticoid insensitivity in the inner ear remain enigmatic. Recent studies indicate that HDAC2 may play a critical role in restoring glucocorticoid-sensitivity^9^. Under normal conditions, glucocorticoids bind to and activate cognate glucocorticoid receptors (GRs). The activated GRs then interact with co-repressor molecules and recruit HDAC2 to attenuate NF-κB-associated co-activator activity to repress transcription of inflammatory genes through active deacetylation of their genomic loci. Under pathologic conditions, however, oxidative stress and inflammation reduce HDAC2 activity and increase its degradation by activating phosphoinositide 3-kinase (PI3K) or acting through the formation of peroxynitrite, which may contribute to glucocorticoid resistance^12, 29, 30^. LPS can induce inflammation and oxidative stress in the cochlea^31^. We hypothesized that LPS would reduce HDAC2 expression in the cochlea to induce glucocorticoid resistance. The current results support our hypothesis. Compared with the untreated LPS-infusion group, the threshold shifts were lower after systemic administration of DEX alone (Fig. 1). Consistent with these ABR results, the degree of pathological changes in the cochlea was reduced by the glucocorticoid treatment (Fig. 7). We also observed lower HDAC2 levels in the cochlear tissue of guinea pigs treated with LPS compared with the AP surgical control group (Fig. 5). Consistent with our results, reduced HDAC2 activity and levels were observed in the lungs of rats in a bronchopulmonary dysplasia animal model by injecting LPS into the amniotic cavity^32^. Correlation analyses provided stronger evidence that HDAC2 is associated with glucocorticoid sensitivity, as there was a negative correlation between the expression levels of HDAC2 in the cochlea and ABR threshold shifts at 8, 16 and 32 kHz (Fig. 6).

Theophylline, a bronchodilator, has been used to treat COPD for more than 50 years^33^. It directly inhibits phosphodiesterases (PDEs) and antagonizes adenosine receptors at relatively high concentrations^34^. However, theophylline at lower concentrations has been shown to elicit an anti-inflammatory effect^35^. Low concentrations of theophylline have also been shown to restore glucocorticoid sensitivity by enhancing HDAC2 activity through inhibition of PI3Kδ, which is independent from PDE inhibition and adenosine antagonism^35, 36^. Thus, these potential targets of theophylline need to be examined in the cochlea in future studies. Interestingly, treatment with AMI alone showed a protective effect against hearing loss but did not significantly change HDAC2 levels in the cochlea (Fig. 5). However, significant hearing improvement, reduced spiral ganglion and stria vascularis degeneration, and increased HDAC2 levels were observed in the animals treated with a combination of DEX and AMI, indicating a synergistic protective effect between AMI and DEX. Although AMI has been shown to enhance HDAC2 activity in chronic obstructive pulmonary disease^36^, treatment with AMI alone did not change HDAC2 levels in the cochlea. An alternative explanation is that AMI may restore glucocorticoid sensitivity in the cochlea through different subunits of HDAC or different pathways. Some studies reported that the combination of AMI and corticosteroid treatment could inhibit inflammatory damage by increasing H_2_S levels, a new and important regulator of inflammation^37^. Further studies, i.e. inflammatory responses in the cochlea after LPS infusion and AMI/DEX treatment^38^, are needed to better understand these mechanisms. The results presented herein also suggest that the low AMI dose administered through the I.P. injection also up-regulated HDAC2 and restored therapeutic steroid responses in the cochlea as in the respiratory system^35, 39, 40^. However, the optimal therapeutic concentration of AMI in the cochlea after I.P. injection needs to be further evaluated in the future.

This study provides evidence that AMI can restore glucocorticoid sensitivity, which may offer a new avenue for treating patients with hearing and vestibular disorders whom are refractory to glucocorticoids.

## Materials and Methods

### Statement of ethics of animal care and use

All animal protocols were approved by the Medical Animal Care and Welfare Committee of Nanjing Drum Tower Hospital, Medical School of Nanjing University, China (20140403). All animal procedures were carried out in accordance with the U.S. National Institute of Health guidelines, including “Principles for Use of Animals” and “Guide for the Care and Use of Laboratory Animals”.

### Animals, cochleostomy, and drug administration

In the present study, we used direct cochlear LPS infusion to induce severe hearing loss in guinea pigs as a means of investigating cochlear HDAC2 levels and the protective efficacy of glucocorticoid and AMI treatment in this animal model^16^. AMI has been shown to restore glucocorticoid sensitivity by enhancing HDAC2 activity in chronic obstructive pulmonary disease^36^. Fifty guinea pigs with body weights between 250 to 350 g were used in this study. All experiments were performed in accordance with the Medical Animal Care and Welfare Committee of Nanjing Drum Tower Hospital, Medical School of Nanjing University, China. All guinea pigs were screened by ABR, and only guinea pigs with normal hearing were used. These animals were randomly divided into five groups: control/ AP group (n = 10, AP only cochlear infusion); LPS only group (n = 10, LPS cochlear infusion without any other injections); LPS + DEX group (n = 10, LPS cochlear infusion plus intraperitoneal injection of DEX); LPS + AMI group (n = 10, LPS cochlear infusion plus intraperitoneal injection of AMI); LPS + DEX + AMI group (n = 10, LPS cochlear infusion plus intraperitoneal injection of AMI and DEX). Two doses of DEX (1 mg/kg) and/or AMI (20 mg/kg) were administered 30 min pre-surgery and 24 hours after surgery. The dose of AMI used in this study was selected on the basis that it had been previously been shown to up-regulate HDAC2, restore steroid responses, and inhibit inflammatory lung injury induced by LPS^33, 39, 40^. The body temperature was maintained at 38 °C, using an electric heating pad during surgery. The mastoid bulla was opened by a postauricular incision to allow visualization of the round window and the basal turn of the cochlea, using a surgical microscope. A hole was made in the basal turn 2 mm away from the rim of the round window, using an electric drill. With the aid of a stereotaxic apparatus and a syringe pump (WPI, USA), a 32 g needle was inserted into the hole to infuse 5 μL of AP (NaCl 145 mM, KCl 2.7 mM, MgSO4 2.0 mM, CaCl2 1.2 mM and HEPES, C8H18N2O4S 5.0 mM)^41^ at a rate of 50 nL/s in both ears in the AP control group, and 5 μL of LPS (Sigma, America) at a concentration of 5 mg/mL in AP in all others groups. The hole in the basal turn of the cochlea was sealed with a small piece of muscle after the infusion. The skin incision was sutured. All surgeries were strictly conducted in compliance with aseptic principles. ABR thresholds were recorded 48 hours after surgery in all guinea pigs.

### ABR tests

ABRs were measured in all guinea pigs used in the present before and 48 hours after surgery. ABRs were assessed in both ears for each animal. All animals were anesthetized with 1% sodium pentobarbital (32–35 mg/kg). After the anesthetic induction, body temperatures were maintained at 38 °C, using an electric heating pad. The recording electrode was placed subcutaneously at the vertex, while the ground and reference electrodes were inserted into the pinnas, respectively. All stimuli were generated by TDT system 3 SigGen software (RZ6, Tucker-Davis Technology, Alachua, FL, USA). Each tone burst was 10 ms in duration with a 5 ms rise/fall time. Stimuli were presented at a rate of 21.1/s. Signals were routed to a speaker (TDT Model MF1) positioned in the ear canal, 1024 sweeps were averaged at each stimulus level. The level of the signal was decreased in 5 dB steps from 90 dB SPL to 5 dB SPL. The ABR threshold was defined as the lowest intensity that a reproducible wave III could be recorded. Four frequencies (4, 8, 16 and 32 kHz) were tested. The average threshold shifts were averaged from both ears in all animals.

### HDAC2 immunolabeling in the cochlear basilar membrane

The distribution of HDAC2 has not been examined in the cochleae of guinea pigs^42, 43^. In the present study, we examined HDAC2 distribution in the guinea pig cochleae, using cochlear basilar membrane stretched preparations and cochlear cryosections (see below). After postsurgical ABR recordings, two animals (four cochleae) in each group were randomly selected for cochlear basilar membrane preparations for HDAC2 immunolabeling. These animals were euthanized, and the temporal bones were removed quickly. The apexes were gently opened with a 32 g needle; the stapes and the round window membranes were removed with forceps; and the cochleae were perfused with cold 4% paraformaldehyde (PFA) in PBS through the round windows and fixed in the same fixative overnight at 4 °C. For cochlear basilar membrane stretched preparations, the lateral bony shell of the cochlea was removed with fine steel needles. Basilar membranes were carefully peeled off, and Reissner’s membrane and the tectorial membrane were removed simultaneously. After rinsing with PBS three times, basilar membranes were incubated with 5% normal goat serum in PBS for 30 min to block non-specific binding of secondary antibody, followed by incubating with rabbit anti-HDAC2 monoclonal antibody (Abcam, Cambridge, UK) at a dilution of 1:1000 overnight at 4 °C. Tissues were then washed three times with PBS. A secondary antibody, goat anti-rabbit IgG (Alexa Fluor®555, Abcam, Cambridge, UK) was used in 1:1000 in dark for 1 hour. Fluorescein isothiocyanate (FITC)-phalloidin (cytoskeleton, USA) at a dilution of 1:500 in PBS was used to stain the stereocilia bundles on hair cells. 4′,6-diamidino-2-phenylindole (DAPI) in PBS was used for nuclear staining. The tissues were whole mounted on slides with Cell-Tak (BD Bioscience, America) and examined with a Leica fluorescence microscope.

### HDAC2 immunolabeling on cochlear cryosections

After post-surgical ABR recordings, the left cochleae (n = 3) from three randomly-selected animals were removed quickly and fixed in 4% PFA overnight. The right cochleae (n = 3) were used for HE staining (see the following). After decalcification with 10% ethylene diamine tetraacetic acid (EDTA) solution for four weeks, fixed cochleae were cryoprotected in 30% sucrose solution in PBS, and then transferred into cryo-molds filled with OCT embedding compound. The cochleae in the molds were frozen at −80 °C for overnight and then sectioned at a thickness of 8 μm with a cryotome (Leica, Germany). Immunofluorescence staining was conducted on these sections, using the same immunostaining protocol used for the whole mount basilar membrane (detailed above).

### Evaluation of HDAC2 levels in the cochlea by ELISA

After post-surgical ABRs recordings, three animals (six cochleae) in each group were euthanized, and the temporal bones were removed quickly. The lateral bony shells of the cochleae were removed with fine steel needles, and the cochlear soft tissues, including the basilar membrane, the spiral ligament, and the stria vascularis, were dissected out and homogenized in 1.5 mL EP tubes. Cochlear nuclear protein extracts were prepared using a Nuclear and Cytoplasmic Protein Extraction Kit (Beyotime Biotechnology, China). Protein concentrations were determined, using a Bicinchoninic Acid assay kit (Beyotime Biotechnology, China). HDCA2 levels were then quantified, using ELISA with EpiQuik HDAC2 Assay kit (Epigentek, Brooklyn, NY). In this assay, the protein concentrations were adjusted to 0.8 µg/µL with HDAC assay buffer and stably coated on the strip wells, blocking for 45 min. HDAC2 was recognized by a high-affinity antibody (provided by the kit mentioned above). The amount of HDAC2 was quantified through an HRP conjugated secondary antibody and color development system. The results were expressed as absorbance units at 450 nm.

### Histopathological analyses in the cochlea

The right cochleae (n = 3) from three animals in each group were used to examine the histopathological changes after LPS treatment by HE staining. Cochleae from each group were decalcified with EDTA, dehydrated with ethanol and embedded in paraffin. Five µm midmodiolar sections were cut, mounted on glass slides and stained with hematoxylin and eosin. The sections were examined with a light microscope.

All spiral ganglion cells with integrated nuclei within each profile of Rosenthal’s canal from base to apex of the cochlea were counted. The size of the ganglion was measured and spiral ganglion cell densities were calculated (number of cells/10,000 μm^2^). The width of the stria vascularis was measured in the middle turns of each cochlea with the aid of ImageJ software. The mean value from three sections from each cochlea was used to define the width of the stria vascularis.

### Statistical analysis

All variables were expressed as means ± standard deviation (SD) and analyzed, using the SPSS 19.0 statistical software package. Differences among the five experimental groups were analyzed by one-way analysis of variance (ANOVA), followed by least-significant differences (LSD) *post hoc* tests to determine differences between groups. A *p* value of less than 0.05 was considered to be statistically significant.
